# Quadriceps strength is negatively associated with knee joint structural abnormalities—data from osteoarthritis initiative

**DOI:** 10.1186/s12891-022-05635-9

**Published:** 2022-08-17

**Authors:** Ze Gong, Jia Li, Zijun He, Shilin Li, Peihua Cao, Guangfeng Ruan, Yan Zhang, Qing Zeng, Rong Chen, Peng Zheng, Tao Fan, Yijin Zhao, Pengcheng Lu, Zhaohua Zhu, Guozhi Huang

**Affiliations:** 1 Department of Rehabilitation Medicine, Zhujiang Hospital, Southern Medical University, Guangzhou, China; 2 School of Rehabilitation Medicine, Southern Medical University, Haizhu District 510280 Guangzhou, China; 3 Division of Orthopaedic Surgery, Department of Orthopaedics, Nanfang Hospital, Southern Medical University, Guangzhou, Guangdong China; 4 Clinical Research Centre, Zhujiang Hospital, Southern Medical University, Haizhu District 510280, Guangzhou, Guangdong China; 5 Clinical Research Centre, School of Medicine, Guangzhou First People’s Hospital, South China University of Technology, Guangzhou, China

**Keywords:** Osteoarthritis, Quadriceps strength, Cartilage damage, Bone marrow lesions, Effusion-synovitis, Hoffa-synovitis

## Abstract

**Objective:**

The aim of this study was to explore the longitudinal associations between baseline quadriceps strength and knee joint structural abnormalities in knee osteoarthritis (KOA).

**Methods:**

This study is a longitudinally observational study based on Osteoarthritis Initiative (OAI) cohort, including men and women aged 45–79. Quadriceps strength was measured by isometric knee extension testing at baseline. Knee joint structural abnormalities, including cartilage damage, bone marrow lesions (BMLs), effusion-synovitis and Hoffa-synovitis, were evaluated by Magnetic Resonance Imaging Osteoarthritis Knee Score (MOAKS) at baseline and 1-year follow-up. Generalized estimating equations were employed to examine the associations between quadriceps strength and knee structural abnormalities. All analyses were stratified by sex.

**Results:**

One thousand three hundred thirty-eight participants (523 men and 815 women) with a mean age of 61.8 years and a mean BMI of 29.4 kg/m^2^ were included in this study. For men, no significantly longitudinal association of quadriceps strength with structural abnormalities was detected. In contrast, quadriceps strength was significantly and negatively associated with changes in cartilage damage and BMLs in lateral patellofemoral joint (PFJ) (cartilage damage: *OR*: 0.91, 95% CI 0.84 to 0.99, *P* = 0.023; BMLs: *OR*: 0.85, 95% CI 0.74 to 0.96, *P* = 0.011) and effusion-synovitis (*OR* = 0.88, 95% CI 0.78 to 0.99, *P* = 0.045) among females longitudinally. Higher quadriceps strength was significantly associated with less progression of lateral PFJ cartilage damage, BMLs and effusion-synovitis in females.

**Conclusions:**

Higher quadriceps strength was associated with changes in cartilage damage and BMLs within the lateral PFJ and effusion-synovitis among females, suggesting the potential protective role of quadriceps strength on joint structures in women.

## Introduction

Knee osteoarthritis (KOA) is characterized by degradation of periarticular cartilage damage, subchondral bone marrow lesions (BMLs) and synovitis﻿ [1], affecting approximately 250 million individuals worldwide [2]. With an aging population coupled with rising obesity rates, there is an urgent need for effective therapy to modify the onset or progression of structural damage in KOA. However, treatment for KOA is restricted to improving symptoms and functions, and there is no effective disease modifying osteoarthritis drugs (DMOADs) to reverse structural lesions in the clinic [3]. Thus, it is essential to identify the protective factors for KOA structural degeneration.

Quadriceps strength has been suggested as a protective factor for KOA, as higher quadriceps strength is commonly associated with delaying KOA progression [3, 4]. Some authorities (such as American College of Rheumatology and Osteoarthritis Research Society International) have strongly recommended quadriceps strength training as the first-line treatment for KOA patients [5–8]. However, how quadriceps protects against the development of KOA is largely unknown. Both biomechanical and biochemical pathways are considered to be involved. Quadriceps, served as knee stabilizer and shock absorber, helps to maintain the joint mechanical environment and dissipates harmful loads on the knee [9]. Besides, quadriceps is also known to produce various myokines, such as cytokines, peptides and growth factors, and thus may crosstalk with knee joint structures at a molecular level [10–12].

The relationships between quadriceps and other knee joint structural abnormalities need to be figured out to improve the understanding of its protection mechanism. Quadriceps strength plays an essential role in the development of KOA. The associations of quadriceps strength with different KOA-related structural abnormalities have been separately studied in the last decades, with inconsistent results [13–16]. Comprehensive studies are still needed to approach the consensus. For cartilage, no firm conclusions can be drawn about the effects of quadriceps on the cartilage damage, regardless of patellofemoral joint (PFJ) or tibiofemoral joint (TFJ) cartilage damage [13–15]. A few studies also explore the association with BMLs, but found limited evidence for a negative association of quadriceps strength with BMLs in PFJ [14, 16]. These studies used a cross-sectional design, thus large-scale longitudinal studies are lacking. Moreover, we noticed that seldom studies concentrated on the knee joint inflammation, such as effusion-synovitis and Hoffa-synovitis, an essential structure catching increasing attention in the KOA study. It follows, therefore, that the relationship between quadriceps strength and knee joint structural abnormalities is required investigation.

The purpose of this study was to examine the cross-sectional and longitudinal associations between baseline quadriceps strength and joint structural abnormalities in participants with or at risk of KOA. We hypothesized that higher baseline quadriceps strength was negatively associated with changes in structural abnormalities both in cross-sectional and longitudinal analyses.

## Methods

### Participants

The Osteoarthritis Initiative (OAI) is a public available, multi-center, prospective cohort study, investigating the risk factors for onset and progression of KOA. OAI comprised 4796 men and women aged 45–79 years with or at risk of KOA and collected their information including clinical, biochemical and imaging measurements. Specific inclusion and exclusion criterions have been reported elsewhere previously [17]. Recruiting and data collection occurred in 5 clinical sites (Maryland/Johns Hopkins University, Memorial Hospital, University of Pittsburgh, and Ohio State University). This study was approved by the Institutional Review Board at each OAI site and all subjects gave informed consent.

**Fig. 1:**
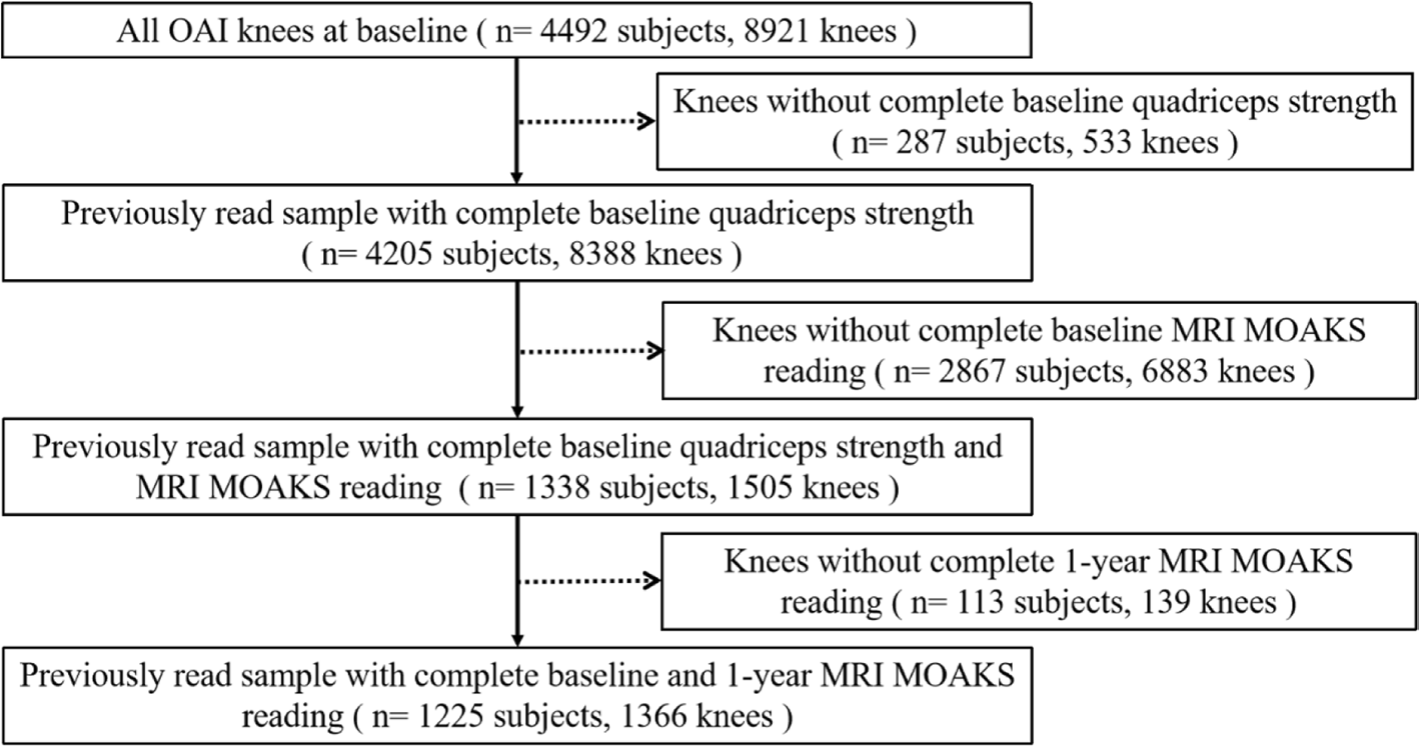
Flow chart showing participant selection from the Osteoarthritis Initiative (OAI). MRI: Magnetic Resonance Imaging; MOAKS: Magnetic Resonance Imaging Osteoarthritis Knee Score. There exists the possibility that two knees of the same participant could be involved in this study

At baseline, 1338 participants (1505 knees) were included from OAI, with complete records of quadriceps strength and knee structural measurements, and 1225 participants (1366 knees) were left at 1-year follow-ups. There exists the possibility that two knees of the same participant could be involved in this study. The flow chart on measurement of key variables is shown in Fig. 1.

The demographic data contained information, including age, sex, race, body mass index (BMI), radiological severity, injury history and surgery history at baseline. At enrolment, clinical data, including age, sex, race and BMI, was collected. Meanwhile, radiological severity was assessed according to Kellgren-Lawrence (KL) grading system (0 to 4). Knee injury was evaluated by asking the participants whether they have ever injured their knee (s) badly enough to limit their ability to walk for at least a week. Knee surgery was defined as a history of any knee surgery, such as arthroscopy, ligament repair and meniscectomy. Further details of all these data, along with quadriceps strength and joint structures were derived from a fully OAI public database (https://nda.nih.gov/oai).

### Quadriceps strength assessment

In current study, maximal isometric quadriceps strength was derived from OAI database at baseline. Trained and certified OAI personnel use a Good Strength Chair (Metitur Oy) to evaluate the quadriceps strength, which has published reliability and validity of this test [18]. OAI subjects were seated upright in the chair, with their legs placed in 60° flexion, and their thighs and pelvises were fixed by straps. A load cell was secured to the test leg, 2 cm proximal to the individual’s calcaneus. To warm up and get familiarized with the testing procedure, participants were instructed to exert 50% effort in first two practice sessions. Then, participants conducted three 100% voluntary isometric contraction on each knee and the highest value was recorded for analyses. To normalize maximum strength with the most appropriate scaling to body weight, we used torque (Nm/kg) to represent maximum isometric strength.

### MRI assessment

Three T knee images of each knee were acquired from 4 OAI clinical sites, using identical MRI systems (Siemens, Erlanger, Germany). Details of OAI pulse sequence protocol and parameters have been published elsewhere [19]. All these MRIs were evaluated by experienced musculoskeletal radiologist (AG and FWR), who were blinded to clinical characteristics. Magnetic Resonance Imaging Osteoarthritis Knee Score (MOAKS) system was adopted to semi-quantitatively assess knee structures [20].

Cartilage damage at baseline and follow-up were evaluated at the medial tibiofemoral (MTF), lateral tibiofemoral (LTF), medial patellofemoral (MPF) and lateral patellofemoral (LPF) compartments as previously described [21]. Cartilage damage size was used to represent the extent of cartilage damage and graded as follows: 0 = normal, 1 = small (< 10% area damaged), 2 = medium (10–75% area damaged) and 3 = large (> 75% area damaged) [21]. Cumulative scores for the size of cartilage damage were calculated for MTF (0 to 15), LTF (0 to 15), MPF (0 to 6) and LPF (0 to 6) joint and changes in cartilage damage were calculated as: (follow-up score − baseline score).

BMLs at baseline and follow-up were scored in the same subregions: 0 = normal, 1 = small (< 33% area damaged), 2 = medium (33–66% area damaged) and 3 = large (> 66% area damaged) [21]. Cumulative scores for the size of BMLs were calculated for MTF, LTF, MPF and LPF joint and changes in BMLs were calculated as: (follow-up score − baseline score).

Effusion-synovitis was graded from 0 to 3 in terms of the estimated maximum distention of the synovial cavity as follows: 0 = normal, 1 = small (< 33% maximum distention), 2 = medium (33–66% maximum distention) and 3 = large (> 66% maximum distention) [20]. Hoffa-synovitis was graded from 0 to 3 in terms of the estimated hyperintensity alteration area (within infrapatellar fat pad area) as follows: 0 = normal, 1 = small (< 33% infrapatellar fat pad area), 2 = medium (33–66% infrapatellar fat pad area) and 3 = large (> 66% infrapatellar fat pad area) [20]. We used the assessments of effusion-synovitis and Hoffa-synovitis from the subject knee at each visit, while changes were calculated as: (follow-up score − baseline score).

According to previous studies, all of the above measures showed good (0.61–0.8) or near-perfect (0.81–1.0) agreement [22].

### Statistical analysis

All analyses were stratified by sex, as there are differences in quadriceps strength and KOA development in men and women. The analyses of knee characteristics are descriptive, with percentages for classification variables and mean (SD) for continuous variables. Normality was analyzed by Shapiro‐Wilk normality test and Levene’s test was used to examine the homogeneity of variance. Independent sample t test and Mann–Whitney U test were used to compare the continuous variables between sexes. Chi-square tests were used for the analysis of classification variables. Considering that dependent variables are ordinal and correlations between two knees of the same participant, General Estimating Equation (GEE) using a Gaussian distribution was employed to evaluate the associations of baseline quadriceps strength with knee joint structural abnormalities at baseline and changes in joint structural abnormalities, such as cartilage damage, BMLs, effusion-synovitis and Hoffa-synovitis, after adjustment for age, BMI, race, KL grade, injury, surgery and baseline structural abnormalities (only for longitudinal analyses). For all regression analyses, *β* coefficients and *P* values were used. In addition, Stata 16.0 (Stata Corp) was used to carry out all statistical analyses and *P* < 0.05 was set as significance.

## Results

### Demographic and clinical characteristics of the OAI subjects studied

Table 1 presents the baseline characteristics of participants and knees. 1338 participants (523 men and 815 women) and 1505 knees (584 men and 921 women) were included in this study. 1505 knees (584 men and 921 women) were included in this study. The mean ± SD age of the participants (*n* = 1338) was 61.8 ± 8.8 years; most of the subjects were overweight (mean ± SD BMI 29.4 ± 4.7 kg/m2). Men and women were similar in terms of age, BMI, effusion-synovitis, cartilage damage and BMLs in the LTF joint. However, women had lower quadriceps strength, cartilage damage and BMLs score in the MTF joint and higher cartilage damage and BMLs score in the PFJ than men. Additionally, the proportion of women with injury or surgery history is lower compared to men. KL grade and Hoffa-synovitis grade distribution was significantly different between men and women.

**Table 1:** Baseline characteristics of the participants and the knees

Characteristics	**Men**	**Women**	***P***
**Participants**^**a**^			
Race (%)	88.5	80.0	**0.001**
BMI (kg/m^2^)	29.4 ± 3.9	29.4 ± 5.1	0.688
Age (years)	61.9 ± 9.0	61.7 ± 8.7	0.589
**Knees**^**b**^			
KL grade, 0/1/2/3/4, (%)	16.4/24.3/19.6/26.0/13.8	16.5/31.5/26.1/18.5/7.4	** < 0.001**
Injury (%)	40.0%	28.2%	** < 0.001**
Surgery (%)	25.9%	12.5%	** < 0.001**
Quadriceps strength (Nm/kg)	1.5 ± 0.5	1.1 ± 0.4	** < 0.001**
Cartilage damage			
MTF (range 0 to 15)	4.0 ± 4.0	2.6 ± 3.3	** < 0.001**
LTF (range 0 to 15)	1.7 ± 2.7	1.8 ± 2.7	0.480
MPF (range 0 to 6)	1.9 ± 1.5	2.5 ± 1.5	** < 0.001**
LPF (range 0 to 6)	1.5 ± 1.8	1.8 ± 1.9	**0.001**
BMLs			
MTF (range 0 to 15)	1.8 ± 2.6	1.0 ± 2.0	** < 0.001**
LTF (range 0 to 15)	0.6 ± 1.5	0.5 ± 1.5	0.287
MPF (range 0 to 6)	0.6 ± 0.9	0.9 ± 1.1	** < 0.001**
LPF (range 0 to 6)	0.8 ± 1.3	1.0 ± 1.4	**0.008**
Synovitis			
Effusion-synovitis, 0/1/2/3, (%)	41.8/34.6/17.5/6.2	43.9/38.2/13.2/4.7	0.059
Hoffa-synovitis, 0/1/2/3, (%)	34.9/49.5/13.7/1.9	45.0/47.0/7.8/0.2	** < 0.001**

Race, white people percentage, *BMI* Body mass index, *KL* Kellgren-Lawrence, *MTF* Medial tibiofemoral, *LTF* Lateral tibiofemoral, *MPF* Medial patellofemoral, *LPF* Lateral patellofemoral, *BMLs* Bone marrow lesions, Effusion-synovitis, graded as 0/1/2/3 according to MOAKS system; Hoffa-synovitis, graded as 0/1/2/3 according to MOAKS system; MOAKS, magnetic resonance imaging Osteoarthritis Knee Score

Bold denoted statistical significance (*P* < 0.05)

^**a**^ For all, *n* = 1338; for men, *n* = 523; for women, *n* = 815

^**b**^ For all, *n* = 1505; for men, *n* = 584; for women, *n* = 921

### Associations of baseline quadriceps strength with baseline and changes in knee cartilage damage

Cross-sectional and longitudinal associations between baseline quadriceps strength and cartilage damage are presented at Table 2. For males, at baseline, quadriceps strength was cross-sectionally associated with cartilage damage at LTF and LPF sites (at LTF: *OR* = 0.54, 95% CI [0.34, 0.85], *P* = 0.008; at LPF: *OR* = 0.67, 95% CI [0.49, 0.91], *P* = 0.011). No significant association of quadriceps strength with worsening of cartilage damage in men was detected. For females, quadriceps strength was significantly and negatively associated with LPF joint cartilage damage in both cross-sectional and longitudinal analyses (cross-sectionally: *OR* = 0.40, 95% CI [0.27, 0.58], *P* < 0.001; longitudinally: *OR* = 0.91, 95% CI [0.84, 0.99], *P* = 0.023). So higher quadriceps strength was associated with less cartilage damage in females rather than males, especially in LPF.

**Table 2:** Associations of baseline quadriceps strength with baseline and changes in cartilage damage

	**Men**		**Women**	
	***OR* (95% CI)**	***P***	***OR* (95% CI)**	***P***
**Baseline cartilage damage**				
MTF	1.32 (0.83, 2.09) ^a^	0.247	1.42 (0.90, 2.22) ^**a**^	0.130
LTF	**0.54 (0.34, 0.85) **^**a**^	**0.008**	0.93 (0.60, 1.45) ^a^	0.759
MPF	0.89 (0.66, 1.19) ^a^	0.424	**0.64 (0.48, 0.85) **^**a**^	**0.002**
LPF	**0.67 (0.49, 0.91) **^**a**^	**0.011**	**0.40 (0.27, 0.58) **^**a**^	** < 0.001**
**Changes in cartilage damage**				
MTF	0.91 (0.81, 1.03) ^b^	0.155	0.97 (0.80, 1.18) ^b^	0.754
LTF	0.96 (0.88, 1.05) ^b^	0.406	1.05 (0.90, 1.22) ^b^	0.529
MPF	0.96 (0.92, 1.01) ^b^	0.165	1.01 (0.97, 1.04) ^b^	0.716
LPF	1.03 (0.97, 1.08) ^b^	0.375	**0.91 (0.84, 0.99) **^**b**^	**0.023**

Generalized estimating equation models were applied

Bold denoted statistical significance (*P* < 0.05)

^**a**^Adjustment for age, race, BMI, KL, injury and surgery at baseline for cartilage damage at baseline

^**b**^Adjustment for age, race, BMI, KL, injury, surgery and cartilage damage score at baseline for changes in cartilage damage over 1 year

### Associations of baseline quadriceps strength with baseline and changes in knee BMLs

Cross-sectional and longitudinal associations between baseline quadriceps strength and BMLs are presented at Table 3. For males, we only found significant association of quadriceps strength with LTF joint BMLs at baseline and there were no significantly longitudinal association between quadriceps strength and changes of BMLs at any site. For females, cross-sectional and longitudinal analyses showed that quadriceps strength was significantly and negatively associated with BMLs in LPF joint (cross-sectionally: *OR* = 0.54, 95% CI [0.40, 0.71], *P* < 0.001; longitudinally: *OR* = 0.85, 95% CI [0.74, 0.96], *P* = 0.011). As shown, higher quadriceps strength was associated with less BMLs in LPF among females.

**Table 3:** Associations of baseline quadriceps strength with baseline and changes in BMLs

	**Men**		**Women**	
	***OR* (95% CI)**	***P***	***OR* (95% CI)**	***P***
**Baseline BMLs**				
MTF	1.35 (0.93, 1.96) ^a^	0.109	0.99 (0.74, 1.32) ^**a**^	0.937
LTF	**0.67 (0.58, 0.89) **^**a**^	**0.005**	0.86 (0.66, 1.13) ^a^	0.279
MPF	1.10 (0.92, 1.31) ^a^	0.285	**0.78 (0.63, 0.97) **^**a**^	**0.028**
LPF	0.87 (0.68, 1.11) ^a^	0.257	**0.54 (0.40, 0.71) **^**a**^	** < 0.001**
**Changes in BMLs**				
MTF	1.05 (0.87, 1.27) ^**b**^	0.584	0.98 (0.77, 1.25) ^**b**^	0.899
LTF	0.93 (0.85, 1.03) ^**b**^	0.161	0.99 (0.87, 1.15) ^**b**^	0.985
MPF	0.99 (0.92, 1.09) ^**b**^	0.982	1.00 (0.90, 1.11) ^**b**^	0.989
LPF	0.94 (0.85, 1.04) ^**b**^	0.241	**0.85 (0.74, 0.96) **^**b**^	**0.011**

Generalized estimating equation models were applied

Bold denoted statistical significance (*P* < 0.05)

^**a**^Adjustment for age, race, BMI, KL, injury and surgery at baseline for bone marrow lesions at baseline

^**b**^Adjustment for age, race, BMI, KL, injury, surgery and bone marrow lesions score at baseline for changes in bone marrow lesions over 1 year

### Associations of baseline quadriceps strength with baseline and changes in synovitis

Cross-sectional and longitudinal associations of baseline quadriceps strength with Hoffa-synovitis and effusion-synovitis are presented at Table 4. For males, no significant association between quadriceps strength and synovitis was observed. For females, at baseline, quadriceps strength was significantly associated with Hoffa-synovitis (*OR* = 0.88, 95% CI [0.78, 0.99], *P* = 0.044). Longitudinally, there existed significant association of baseline quadriceps strength with effusion-synovitis (*OR* = 0.88, 95% CI [0.78, 0.99], *P* = 0.045) but not with Hoffa-synovitis. As we can see, higher quadriceps strength was associated with less effusion-synovitis in females.

**Table 4:** Associations of baseline quadriceps strength (Nm/kg) with baseline and changes in synovitis

	**Men**		**Women**	
	***OR* (95% CI)**	***P***	***OR* (95% CI)**	***P***
**Baseline synovitis**				
Effusion-synovitis	0.93 (0.80, 1.08) ^**a**^	0.322	0.94 (0.80, 1.10) ^**a**^	0.443
Hoffa-synovitis	0.92 (0.85, 1.05) ^**a**^	0.208	**0.88 (0.78, 0.99) **^**a**^	**0.044**
**Changes in synovitis**				
Effusion-synovitis	0.91 (0.82, 1.01) ^**b**^	0.085	**0.88 (0.78, 0.99)** ^b^	**0.045**
Hoffa-synovitis	0.99 (0.94, 1.04) ^**b**^	0.621	0.99 (0.94, 1.05) ^**b**^	0.729

Generalized estimating equation models were applied

Bold denoted statistical significance (*P* < 0.05)

^**a**^Adjustment for age, race, BMI, KL, injury and surgery at baseline for synovitis at baseline

^**b**^Adjustment for age, race, BMI, KL, injury, surgery and synovitis score at baseline for changes in synovitis over 1 year

## Discussion

Although there have been several studies using quadriceps strength as a surrogate for predicting the progression of KOA, this is the first comprehensive cohort study to investigate the associations of baseline quadriceps strength with changes in knee joint structural abnormalities, including cartilage damage, BMLs, effusion-synovitis and Hoffa-synovitis, among men and women. We found that higher quadriceps strength was negatively associated with changes of cartilage damage and BMLs within lateral PFJ and effusion-synovitis in females. No significant longitudinal association between quadriceps strength and structural abnormalities was detected in males. Our findings suggest that quadriceps strength may play a more important role in protecting joint structures in females, especially for PFJ and effusion-synovitis, compared to males.

Knee cartilage damage is a crucial feature of KOA. Abnormal overloading and inflammatory factors have been related to cartilage damage [23, 24]. Quadriceps is the main absorber of harmful loading and its secretion, myokines (e.g., insulin-like growth factor 1 and Irisin), may have chondroprotective effects [10, 11]. Hou et al*.* [25] conducted a meta-analysis concerning the association with cartilage damage, suggesting there existed a trend that lower quadriceps strength could increase the risk of cartilage damage in both TFJ and PFJ. However, this meta-analysis did not take the effect of gender into consideration. As known, both quadriceps strength and KOA development are obviously different among males and females. Our study conducted sex-stratified analyses and reported that quadriceps strength was significantly associated with focal worsening cartilage damage in lateral PFJ in females over 1 year. Similar to our study, Culvenor et al*.* [26] also focused on the sex-specific relation of quadriceps strength to worsening of cartilage damage and found that lower quadriceps strength might increase the risk of lateral PFJ cartilage damage in women. Nevertheless, Chin et al*.* [15] found that lower quadriceps strength could predict cartilage damage in medial TFJ rather than in LPF joint over 3 years. These divergent findings are probably attributed to the different study populations and designs. Moreover, there are multi risk factors for the cartilage damage, and the role quadriceps strength plays may vary in the different stages of cartilage damage. Thus, there is a need for studies focused on the different cartilage damage stage in the future.

BMLs are the most common subchondral bone abnormalities in KOA [27]. According to previous studies, BMLs are closely related to KOA symptoms, and can predict joint replacement and cartilage damage [28–30]. Previously, Stefanik et al*.* [14] and Baert et al. [16] conducted cross-sectional studies and reported that quadriceps strength was significantly associated with BMLs in lateral PFJ. However, these two studies were limited by its cross-sectional design, and did not explore the causation between quadriceps weakness and BMLs. Additionally, these two studies did not take the factor of sex into account, which might cause bias in their final results. Our study firstly reported the significantly longitudinal association of quadriceps strength with worsening of BMLs in lateral PFJ in females, further indicating higher quadriceps strength might play an essential role in the protection of knee joint in females, especially for lateral PFJ. Notably, we must be aware of the short follow-up period of our study, which might influence the confidence to our results. Long-term prospective studies are required to examine the associations between quadriceps strength and BMLs.

Synovitis is a crucial manifestation of KOA. Reflected as synovial membrane thickening or joint effusion [22], synovitis could strongly predict the development of KOA [31]. The use of effusion-synovitis and Hoffa-synovitis as surrogates used to identify joint inflammation in KOA is a commonly deployed method based on non-contrast enhanced MRI [32]. Several studies have reported the significant association between quadriceps strength and effusion-synovitis [16, 33], but their cross-sectional design limited their ability to infer causality. Further, there exists seldom studies concerning about the Hoffa-synovitis. In the current study, we revealed the significant and negative associations between quadriceps strength and changes in effusion-synovitis during the follow-up periods. Our results indicates that quadriceps strength may have a protective effect on effusion-synovitis in females. As reported, effusion synovitis has an impact in the early KOA and may be able to increase risk of cartilage damage [34]. As a precursor of KOA, effusion-synovitis might be more susceptible to be affected by potential risk factor for KOA. Noteworthy, we didn’t find protective effect of quadriceps on Hoffa-synovitis longitudinally. We speculate the possible reason is that quadriceps might have different impact on effusion-synovitis and Hoffa-synovitis due to different assessment location or feature of these two kinds of synovitis. Future clinical trials examining the effect of quadriceps strength training on different kind of synovitis in KOA patients are warranted.

Interestingly, we found that higher quadriceps strength was negatively associated with changes in knee structural abnormalities, such as effusion-synovitis, cartilage damage and BMLs in lateral PFJ among females, but not among males. There are several possible reasons for this discrepancy. Compared with quadriceps strength in males, quadriceps strength in females is lower and more prone to approach the strength threshold needed to protect the knee joint [35]. Apart from strength weakness, narrower step width and wider pelvis [36] in females could also contribute to increased mechanical loading on knees, and all these biomechanical factors might influence the role quadriceps strength plays in female knees, leading to the discordance between men and women.

Quadriceps strength is an important indicator of quadriceps fitness in the clinic. The underlying mechanism of the associations between quadriceps strength and structural abnormalities is unclear, but biomechanical and biochemical pathways have been involved [12]. As a dynamic stabilizer of knee joint, quadriceps may help to reduce intra-articular stress and maintain biomechanical environment [37, 38], and indeed, there have been studies reporting that stable biomechanical environment was associated with knee structural changes [39–41]. It appears that quadriceps could protect joint structures through biomechanical pathways.

Additionally, according to previous studies, quadriceps strength has been suggested to be closely associated with patella trajectory [42] and quadriceps weakness might be unable to sustain a stable environment for PFJ, leading to cartilage damage or BMLs in PFJ. Different with PFJ, our study did not find significant associations between quadriceps strength and changes in TFJ structural abnormalities. This discordance might be attributed to other biomechanical factors, since the other structures within the TFJ like menisci could dissipate the loading which was not assessed in our study. As a whole, the relationship between muscle strength and TFJ structural abnormalities is still complex and more comprehensive studies are needed.

Biochemically, previous studies focusing on quadriceps biochemical and molecular interactions also provided some evidence for its protective effect on knee structures. As quadriceps fitness impaired, quadriceps strength tends to decrease, and accompany with fluctuating inflammation regulators. Myokines (e.g., irisin and interleukin-6) secreted from skeletal muscle are thought to be important regulating regulators in KOA. Cairns et al*.* [10] reported that cytokines and growth factors released from muscle could potentially regulate cartilage damage. He et al*.* [43] reported that Irisin, a kind of myokines, could reduce osteocyte apoptosis and improve the micro-architecture of subchondral bone to attenuate the progression of KOA. Krishnasamy et al*.* [12] reported that muscle might crosstalk with joint structures through anti-inflammatory and pro-chondrogenic mechanisms. Based on the above evidence, we can speculate that quadriceps may also provide effective protection on joint structures through myokine pathway. Further, hormone levels clearly differ between males and females. As reported, hormone have been suggested as the key factor affecting muscle strength and KOA development [44, 45]. Thus, hormone is an important potential confounder for the relationship between quadriceps strength and structural abnormalities and the different results observed between sexes might be partly attributed to it.

The main strength of this study is that we comprehensively explored the cross-sectional and longitudinal associations of quadriceps strength with changes in knee joint structural abnormalities with a large sample size. However, some potential limitations must be acknowledged. First, our results could not necessarily be generalized to other measures of quadriceps strength (e.g., isokinetic), since only isometrical strength was measured in the OAI. Nevertheless, strong correlations between different measures (e.g., isometric, isotonic and isokinetic) indicate similar results would occur regardless of type of strength assessed [46], which might facilitate the generalization of current results. Second, joint structural abnormalities were not measured quantitively. However, we adopted MOAKS with high reliability and validity to evaluate the structural abnormalities, and this scoring system has been applied to a great number of studies. Third, a proportion of participants with histories of knee injury and surgery were involved in our study, though we have adjusted them in statistical models in this study. We failed to further exclude participants with severe histories of knee injury and surgery specifically (e.g., anterior cruciate ligament injury, meniscal injury and anterior cruciate ligament reconstruction), due to the ambiguous description of record in this study. Finally, as an exploratory study, we only investigated the association between quadriceps strength and structural worsening in a short term due to the loss of follow-up in our study. The fluctuation of quadriceps strength was expected to be considered in the future study with longer follow-up.

## Conclusions

This comprehensive study suggests that quadriceps strength plays a protective role in the knee structural abnormalities in females, especially in the lateral patellofemoral joint and effusion-synovitis.

## Data Availability

The datasets generated and/or analyzed during the current study are available from the Osteoarthritis Initiative (OAI) dataset, (https://nda.nih.gov/oai), which is publicly available.

## Abbreviations

KOA: Knee osteoarthritis
BMI: Body mass index
KL: Kellgren-Lawrence
BMLs: Bone marrow lesions
PFJ: Patellofemoral joint
TFJ: Tibiofemoral joint
MTF: Medial tibiofemoral
LTF: Lateral tibiofemoral
MPF: Medial patellofemoral
LPF: Lateral patellofemoral
CI: Confidence intervals
OAI: Osteoarthritis initiative
